# Factors associated with presenteeism due to work-related musculoskeletal disorders

**DOI:** 10.47626/1679-4435-2020-508

**Published:** 2020-12-11

**Authors:** Giselle de Santana Vilasboas Dantas, Jefferson Paixão Cardoso

**Affiliations:** 1 Programa de Pós-Graduação em Enfermagem e Saúde, Universidade Estadual do Sudoeste da Bahia (UESB) - Jequié (BA), Brazil; 2 Núcleo de Estudos em Saúde da População (NESP), Departamento de Saúde II, UESB - Jequié (BA), Brazil

**Keywords:** presenteeism, worker, occupational health, cumulative trauma disorders, occupational diseases

## Abstract

**Background:**

Presenteeism associated to work-related musculoskeletal disorders is an important aspect of occupational health that can reveal important information regarding productivity and quality of life at work.

**Objective:**

To quantify and evaluate the factors associated to presenteeism due to work-related musculoskeletal disorders.

**Methods:**

This is a crosssectional study that used data from the Brazilian National Survey of Health of 2013, involving 847 workers diagnosed with workrelated musculoskeletal disorders.

**Results:**

A multivariate analysis observed an association between presenteeism due to workrelated musculoskeletal disorders and income of a minimum wage or less (prevalence ratio 1.27, 95% confidence interval 1.02-1.60), intense physical activity at work (prevalence ratio 1.22, 95% confidence interval 1.08-1.37), and chronic diseases (prevalence ratio 1.23, 95% confidence interval 1.09-1.38).

**Conclusions:**

Our observations indicated a high incidence of presenteeism, which was associated to sociodemographic and occupational aspects, as well as to the workers’ lifestyle and health conditions.

## INTRODUCTION

Presenteeism is a result of the relationship between an individual, work, and an organization.^1^ It has become an increasing concern among companies and health care professionals due to its negative impact on occupational health,^2^ since it can worsen workers’ health problems and quality of life.^3^ In addition, presenteeism is considered an intermediate phase on the way to absenteeism or sickness leaves.^4^^,^^5^

This phenomenon is defined as the physical presence of the worker at the workplace in spite of a problem that limits work performance, such as a health issue.^3^ That is to say, although the individual is physically at work, he or she cannot fully function,^6^ has a reduced productivity,^7^ and can overload his or her colleagues.^5^

Among many health problems that can lead do presenteeism, work-related musculoskeletal disorders (WMSD) are considered important events in the evaluation of impacts to people’s health.^8^^,^^9^ These injuries can considerably interfere in daily life and work activities, finally resulting in incapacity, additional costs to the company, and loss of productivity or even of the job itself.^9^ In general, presenteeism varies greatly when considering the reasons for maintaining a specific work activity.^10^ However, increases in its incidence have been observed both in population-based surveys^10^ and in specific populations such as health care workers (nurses, physical therapists, and doctors).^7^^,^^11^

Studies that evaluated presenteeism due to musculoskeletal problems^11^^,^^12^ have revealed a disturbing situation, since its high incidence can reflect an increase in WMSD, mainly considering unconfirmed or undiagnosed cases. In addition, the consequences of this type of presenteeism can include not only a reduced work performance, but also impacts on the whole organization.^13^

Presenteeism is a relatively new research topic that requires an attentive and deep study because measuring it is a challenge for organizations: When an individual is affected by a health problem, a reduction in work productivity is not always noticeable. Moreover, in the mid-and long-term, presenteeism can be more harmful to health, productivity, and to the organization itself than absenteeism.^14^

Important elements that favor the occurrence of presenteeism due to WMSD should be explored so as to better comprehend their relationships. Therefore, this study aimed to evaluate aspects associated to the occurrence of presenteeism due to WMSD; for this, we analyzed data presented by the National Survey of Health (PNS), performed in 2013.

## METHODS

This is a cross-sectional study regarding presenteeism due to WMSD in Brazil, in 2013, based on data from the PNS. This survey was performed by the Brazilian Institute of Geography and Statistics (IBGE) and Ministry of Health, along with higher education and research institutes. It consisted of a national household survey that interviewed the population permanently living in a private household. The survey initially chose 81 767 households, of which 62 986 answered to an interview (22% of the households did not respond). The sample was further divided into 3 levels of organization (census sectors, households, and individuals). Field work was performed by IBGE enumerators, supervisors, and coordinators, including interviews, health checks, and collection of biological material. A specific publication described sample size calculation, sampling, and field work in detail.^15^

The final sample of individuals over 18 years old included 60 202 people, of which 847 answered “yes” to the question “Have you ever received a medical diagnosis of WMSD?” and were working during the surveyed period. Our dependent variable was presenteeism due to WMSD, which was evaluated through the following question: “To what degree do WMSD limit your daily activities such as work and household chores?” Possible answers included “they do not,” “a little,” ”moderately,” “intensely,” and “very intensely.” We considered presenteeism when the participant answered that WMSD limited his or her activities in any degree, and excluded cases in which the answer was “they do not.” Another study has also investigated the frequency with which WMSD negatively influence work activities through this question.^16^

Our study also assessed the following sociodemographic factors presented by the survey: sex (male/female), productive age (divided in under 30, 30-59, or over 60 years old), and body mass index (BMI) calculated using weight in kilograms divided by height in meters squared, both measured by the survey team. BMI values were divided into underweight (BMI < 18.5 kg/m^2^), eutrophic (18.5 < BMI < 25 kg/m^2^), and overweight/obese (BMI ≥ 25). Other factors included marital status, divided into having a partner (married) or not (separated, divorced, widow/ widower, single); race/skin color, divided into White and Black/Brown (Yellow, Indigenous, and unknown were removed from our analysis since their absolute frequencies were too low); and schooling, spanning 3 categories: basic education or less, secondary education, or undergraduate education. Incomes were evaluated considering the minimum wage (MW) at the time (R$ 678.00) and were divided into: MW or less, 2 to 3 times the MW, or 3 or more times the MW.

Occupational factors surveyed by this study included the type of employment relationship (public sector, private sector, or no employment contract/job), the number of jobs (1, 2, 3 or more), the presence of a night shift (yes or no), long displacements at work (yes or no), intense physical activity at work (yes or no), and intense physical activity during domestic activities (yes or no). Regarding lifestyle and health conditions, the survey assessed the presence of health insurance (yes or no), regular physical activity (yes or no), and smoking habits (daily, less than daily, or never - later divided into “yes” or “no”). Alcohol consumption was divided into “yes” (once a month or more) or “no” (never or less than once a month). Comorbidities considered the following conditions: high blood pressure, diabetes, hypercholesterolemia, cardiopathies, previous cerebrovascular accident, asthma, musculoskeletal disorders (arthritis, rheumatism, chronic spine disorders), depression, chronic pulmonary obstructive disease, cancer, chronic kidney disease, and other chronic diseases. These comorbidities were assessed using questions regarding the individual’s medical history, which have also been used in other population-based surveys.^17^^-^^19^

Our data analysis was performed in 3 steps. Initially, we performed a descriptive analysis in order to characterize the study population and the occurrence of presenteeism due to WMSD. Subsequently, we verified the factors associated to the outcome (presenteeism due to WMSD) through a bivariate analysis using this outcome and the independent variables. Associations were measured using prevalence ratio (PR) and 95% confidence intervals (95%CI).

We used a multivariate logistic regression for the simultaneous analysis of the variables and presenteeism. Initially, variables with Wald test results of less than 0.20 in the bivariate analysis were selected. The model was evaluated using a backward method and maintaining variables with the smallest Akaike information criterion (AIC), along with those corresponding to the theoretical justification. Adjusted PRs and 95%CIs were then obtained for the remaining variables and a Poisson regression with a robust error variance was used for converting the association measure.^20^ Our final logistic model was evaluated using the Hosmer-Lemeshow test and was graphically verified using the area under the receiver operating characteristic (ROC) curve. The analysis considered the sampling weight of each participant. All analyses were performed using STATA software, version 12.

The PNS was approved by the National Committee of Ethics in Research (CONEP) of the National Health Council (CNS), Ministry of Health (CAAE: 10853812.7.0000.0008), therefore respecting all ethical principles for research involving human beings.

## RESULTS

Among participants with a medical diagnostic of WMSD and working at the time (n = 847), the general prevalence of presenteeism was 59% (n = 500). Female participants presented a higher prevalence of presenteeism due to WMSD (60.4%), and participant age in this group varied from 19 to 83 years old (mean, 41.7; SD, 10.9 years). Among those that were 60 years old or older, the prevalence was 55.6%, while those between 30 and 59 years old showed a prevalence of 60.3% (Table 1).

**Table 1:** Prevalence, prevalence ratio, and 95% confidence interval for presenteeism due to work-related musculoskeletal disor- ders, according to sociodemographic characteristics. National Survey of Health, Brazil, 2013.

Variables	n	P%	PR	95%CI
Sex				
Male	152	56.0	1.00	
Female	348	60.4	1.07	0.95-1.21
Age (years)				
< 30	65	53.8	1.00	
30-59	410	60.3	1.13	0.94-1.35
≥ 60	25	55.6	1.04	0.76-1.42
Schooling				
Basic education or less	205	71.4	1.53	1.28-1.83
Secondary education	194	54.0	1.16	0.96-1.40
Undergraduate education	78	46.4	1.00	
Marital status				
Partner	216	56.9	0.93	0.93-1.05
No partner	284	60.7	1.00	
Race/skin color*				
White	223	54.7	1.00	
Black/Brown	263	63.3	1.15	1.03-1.29
Body mass index				
Underweight	4	57.1	0.94	0.49-1.81
Eutrophic	169	60.4	1.00	
Overweight/obese	226	54.5	0.90	0.79-1.02
Income (times the minimum wage)				
≤ 1	152	67.6	1.48	1.23-1.79
2-3	238	58.0	1.28	1.06-1.54
≥ 3	78	45.3	1.00	
Health insurance				
No	285	64.7	1.22	1.09-1.37
Yes	215	52.8	1.00	

95%CI: 95% confidence interval; P: prevalence; PR: prevalence ratio.

* Yellow and Indigenous populations were excluded from the analyses and corresponded to 5 participants (1%).

The participants with the highest prevalence of presenteeism due to WMSD were those with basic education or less (71.4%), no partner (60.7%), of Black/Brown race/skin color (63.3%), eutrophic (60.4%), earning a MW or less (67.6%), and without health insurance (64.7%).

Characteristics associated to presenteeism due to WMSD were: basic education or less (PR, 1.53; 95%CI 1.28-1.83), Black/Brown race/skin color (PR, 1.15; 95%CI 1.03-1.29), income of a MW or less (PR, 1.48; 95%CI 1.23-1.79) or 2-3 times the MW (PR, 1.28; 95%CI 1.06-1.54), and lack of health insurance (PR,1.22; 95%CI 1.09-1.37) (Table 1).

Table 2 shows the occupational variables, among which the highest prevalence of presenteeism due to WMSD happened with participants with no employment contract (66.5%), who worked night shifts (59.1%), had long displacements at work (61.7%), performed intense physical activity at work (70.4%), and performed intense physical exercise during domestic activities (60.6%). Participants who had 1 job and 3 or more jobs presented similar prevalence (58.8% and 57.1%, respectively).

**Table 2:** Prevalence, prevalence ratio, and 95% confidence interval for presenteeism due to work-related musculoskeletal disor- ders, according to occupational and lifestyle characteristics. National Survey of Health, Brazil, 2013.

Variables	n	P%	PR	95%CI
Type of employment relationship				
Public sector	71	50.7	1.00	
Private sector	272	56.8	1.12	0.93-1.34
No employment contract	131	66.5	1.31	1.08-1.58
Number of jobs				
1	449	58.8	1.00	
2	21	46.7	0.79	0.57-1.09
3 or more	4	57.1	0.97	0.51-1.85
Night shift				
No	78	53.4	1.00	
Yes	396	59.1	1.10	0.93-1.30
Long displacements at work				
No	234	54.8	1.00	
Yes	240	61.7	1.12	1.01-1.26
Intense physical activity at work				
No	322	53.7	1.00	
Yes	152	70.4	1.31	1.16-1.46
Intense physical exercise during domestic activities				
No	340	58.3	1.00	
Yes	160	60.6	1.03	0.92-1.17
Regular physical activity				
No	320	62.9	1.18	1.05-1.34
Yes	180	53.0	1.00	
Smoking habits				
No	423	58.4	1.00	
Yes	77	62.6	1.07	0.92-1.24
Alcohol consumption				
No	357	61.0	1.00	
Sim	143	54.6	0.89	0.78-1.01
Chronic disease				
No	302	53.6	1.00	
Yes	198	69.7	1.29	1.16-1.45

95%CI: 95% confidence interval; P: prevalence; PR: prevalence ratio.

Regarding lifestyle characteristics (Table 2), the highest prevalence of presenteeism due to WMSD happened with participants with no regular physical activity (62.9%), smokers (62.6%), and with a chronic disease (46.6%). Surprisingly, participants who did not drink had a prevalence of 61%. When it came to occupational characteristics, the absence of an employment contract (PR, 1.31; 95%CI 1.08-1.58), long displacements at work (PR, 1.12; 95%CI 1.01-1.25), and intense physical activity at work (PR, 1.31; 95%CI 1.16-1.46) were associated to the outcome. Lifestyle-related variables associated to the outcome according to the bivariate analysis were: no regular physical activity (PR, 1.18; 95%CI 1.05-1.34) and chronic disease (PR, 1.29; 95%CI 1.16-1.45) (Table 2).

The multivariate analysis (Table 3) showed that presenteeism due to WMSD was associated to an income of a MW or less (PR, 1.27; 95%CI 1.02-1.60), intense physical activity at work (PR, 1.22; 95%CI 1.081.37), and chronic disease (PR, 1.23; 95%CI 1.09-1.38). Model diagnostics through the Hosmer-Lemeshow test showed a good fit (p = 0.490) and indicated that the observed frequencies did not correspond to the expected values. The area under the ROC curve was 0.649, indicating a reduced discrimination capacity.

**Table 3:** Final regression model with prevalence ratios and 95% confidence intervals of presenteeism due to work-related musculoskeletal disorders, according to selected variables. National Survey of Health, 2013.

Variables	PR	95%CI
Schooling		
Basic education or less	1.20	0.96-1.48
Secondary education	0.97	0.80-1.23
Undergraduate education	1.00	
Income (times the minimum wage)		
≤ 1	1.27	1.02-1.60
2-3	1.18	0.95-1.45
≥ 3	1.00	
Intense physical activity at work		
Yes	1.22	1.08-1.37
No	1.00	
Chronic disease		
No	1.00	
Yes	1.23	1.09-1.38

95%CI: 95% confidence interval; P: prevalence; PR: prevalence ratio.

## DISCUSSION

This study investigated presenteeism due to WMSD and revealed a worrying situation due to the high prevalence observed in our and other studies.^10^^,^^11^^,^^21^ This outcome considerably affects the worker, indicating an impossibility of performing work activities adequately. The negative impact on workers’ health and on their surroundings can become evident^5^ when the causes of presenteeism are not removed or reduced, since injuries are then aggravated and the quality of work is reduced. Among the main reasons that may cause the worker to not seek assistance are fear of unemployment,^22^ lack of opportunities, a high tolerance to poor work conditions, and the feeling of duty regarding one’s tasks.^23^

The signs and symptoms of WMSD that limit or prevent work can contribute to presenteeism. Most times, no concrete diagnosis of WMSD has been performed, exposing workers to situations of skepticism regarding their problem. Workers thus continue to execute their tasks even if they cannot perform them satisfactorily.^24^

The association between low schooling and presenteeism did not corroborate the results obtained by Merril et al.^25^ This study observed higher presenteeism levels among workers with secondary or undergraduate education, probably due to the fact that these workers performed sedentary administrative jobs that could contribute to the outcome. In contrast, outdoor work presented a lower prevalence of presenteeism, but unfortunately these characteristics were not considered in the PNS.

On the other hand, our results may suggest a relationship regarding work activities performed by individuals with low schooling levels, high workloads, and low job control. In these conditions, workers face higher physical demands, which along with work stress and time constraints, could contribute as predictors of presenteeism.^25^^-^^27^ Moreover, it is likely that workers with lower schooling levels perform activities with higher levels of exposure to risk factors for presenteeism.^28^

Black and Brown races/skin colors were associated to presenteeism due to WMSD. This result may indicate unfavorable conditions for this group to seek job adaptation or health care when considering comorbidities^29^; this highlights the deep differences in life and work conditions among these populations.^30^ Lower incomes were also associated to presenteeism due to WMSD. Other evidences confirm this result,^31^ and it is believed that people in this socioeconomical group rely on their income for their basic needs and expenses, thus remaining at work even when sick. Another item associated to our outcome was the lack of health insurance. This condition can contribute to maintaining presenteeism and indicates difficulties both in the availability and access to specialized health care that could reduce or eliminate the negative repercussions of WMSD.

Factors characterizing a physically demanding job (long displacements and intense physical activity at work) were also associated to presenteeism due to WMSD. These are aspects of physical demands of work that can in turn be considered predictors of musculoskeletal disorders.^32^ Employers can thus promote changes in the work environment so that the health and quality of life of employees could be improved,^33^ reducing the effects of these disorders and preventing new health issues.

Lack of regular physical activity was also shown as associated to our outcome, and this result is corroborated by the literature.^22^^,^^25^^,^^34^ Regular physical activity is known to contribute to promoting and restoring health, consequently reducing presenteeism.^35^ In addition, it promotes health and improves quality of life, increasing cardiorespiratory fitness, strength, as well as bone and overall metabolic health.^36^ Therefore, physical inactivity can contribute to the development and worsening of diseases and preexisting conditions and to the occurrence of presenteeism. Physical activity reduces the harmful effects of a tiring work routine, improving self-esteem,^37^ and can neutralize the negative effects of physical inactivity and sedentary work (especially long sitting periods, which have been shown to be an important indicator of cardiovascular and metabolic risk).^38^

Workers with chronic diseases are more susceptible to presenteeism than those with no chronic disease.^39^ The incidence of these events can be more expressive when along with a lack of regular physical activity, mainly when considering functional incapacities.^40^ Therefore, comorbidities are important characteristics in the evaluation of presenteeism due to WMSD, since they can aggravate this condition^41^ and considerably affect quality of life. Our results regarding this association were also confirmed by other studies.^41^

It is important to note that this study has limitations regarding its cross-sectional design, since it does not allow causal inferences. Another limitation considers the healthy worker effect, since workers on leave or that abandoned the job were not considered by this household survey. Moreover, the WMSD diagnostic was self-reported and with no direct evaluation of the participant’s causal nexus, which could confirm or exclude cases. Finally, it was not possible to measure presenteeism in a scalar manner, which could help quantifying it (for example, with instruments used in occupational research).^42^

## CONCLUSIONS

We concluded that the prevalence of presenteeism was high and associated to a basic education or lower schooling, Black/Brown race/ skin color, lower incomes, lack of health insurance and of an employment contract, long displacements and intense physical activity at work, absence of regular physical activity, and chronic disease. On a multivariate analysis, income, intense physical activity at work, and chronic disease were maintained as factors associated to presenteeism due to WMSD. Our findings highlight the importance of studying WMSD and the resulting presenteeism, as well as their impacts on organizations and occupational health, including workers’ quality of life.

Epidemiologic observational and experimental and support systems should recognize presenteeism as studies are extremely important in order to expand the an existing phenomenon in the workplace and develop knowledge on presenteeism and to search for strategies policies that guarantee its identification and the for reducing its occurrence. Moreover, organizations adequate referrals to health care services.
